# Directional hydrophone clusters reveal evasive responses of small cetaceans to disturbance during construction at offshore windfarms

**DOI:** 10.1098/rsbl.2022.0101

**Published:** 2023-01-18

**Authors:** I. M. Graham, D. Gillespie, K. C. Gkikopoulou, G. D. Hastie, P. M. Thompson

**Affiliations:** ^1^ Lighthouse Field Station, School of Biological Sciences, University of Aberdeen, Cromarty, Ross-shire IV11 8YL, Scotland; ^2^ Sea Mammal Research Unit, Scottish Oceans Institute, University of St Andrews, Fife KY16 8LB, Scotland

**Keywords:** evasive response, mitigation measures, acoustic deterrent device, offshore windfarm, passive acoustic monitoring, phonotaxis

## Abstract

Mitigation measures to disperse marine mammals prior to pile-driving include acoustic deterrent devices and piling soft starts, but their efficacy remains uncertain. We developed a self-contained portable hydrophone cluster to detect small cetacean movements from the distributions of bearings to detections. Using an array of clusters within 10 km of foundation pile installations, we tested the hypothesis that harbour porpoises (*Phocoena phocoena*) respond to mitigation measures at offshore windfarm sites by moving away. During baseline periods, porpoise movements were evenly distributed in all directions. By contrast, animals showed significant directional movement away from sound sources during acoustic deterrent device use and piling soft starts. We demonstrate that porpoises respond to measures aimed to mitigate the most severe impacts of construction at offshore windfarms by swimming directly away from these sound sources. Portable directional hydrophone clusters now provide opportunities to characterize responses to disturbance sources across a broad suite of habitats and contexts.

## 1. Introduction

Information on animal movements underpins a wide range of behavioural and ecological questions, including assessing the responses of wildlife to anthropogenic activities [1,2]. Recent advances in tagging have catalysed movement research [3] but methodological and ethical constraints mean that tags cannot be used for many situations or species. Even where feasible, tagging studies are often limited to a few individuals, locations or short periods of time, constraining the predictive power of results or precluding particular perturbations or locations from study.

Passive acoustic monitoring (PAM) is non-invasive and widely used to assess species diversity, distribution and abundance in terrestrial [4] and marine environments [5]. PAM is also increasingly used to examine wildlife responses to anthropogenic activity (e.g. [6,7]). Importantly, these approaches have resulted in significant advances in understanding of marine mammal reactions to impulsive noise such as pile-driving [8,9]. However, broad-scale movement responses and displacement are typically inferred indirectly from the presence or absence of acoustic detections. Critically, these studies may be confounded if responses also involve changes in acoustic behaviour (e.g. [10]). Direct empirical data on movements of individual animals are therefore also required. Recent technological advances in passive acoustic monitoring have produced systems to localize animals in two-dimensional or three-dimensional space, allowing fine-scale movement behaviour to be studied (e.g. [11–13]). However, opportunities to address other ecological, conservation or management questions with these systems are often constrained given their reliance on existing marine infrastructures for power, equipment or attachment (e.g. U.S. Navy Ranges [12] and renewable energy structures [6,11]).

With the rapid expansion of offshore renewable energy developments, the potential for impacts on birds, bats and marine mammals is increasing. Marine mammals are potentially vulnerable to construction and operational noise or collision risk from tidal turbines [14]. Mitigation measures to reduce the risk of death or injury from impulsive construction noise include using acoustic deterrent devices (ADD) to disperse marine mammals prior to pile-driving, and a piling soft start [15]. However, few studies have assessed the efficacy of these mitigation measures, in part due to the difficulty of obtaining movement data at appropriate temporal and spatial scales, but see [16].

We developed a self-contained and portable hydrophone cluster to measure the direction of arrival of detected sounds. Using an array of these clusters, we recorded movement responses of harbour porpoises (*Phocoena phocoena*) during mitigation activity before and after the onset of piling at an offshore windfarm. We measured how porpoises respond to ADD mitigation and the piling soft start by analysing the distributions of bearings to porpoise clicks, and tested the hypothesis that porpoises respond to these mitigation measures by moving away from sound sources.

## 2. Methods

### (a) Directional hydrophone clusters

Each directional hydrophone cluster (hereafter cluster) comprised: a stainless-steel platform housing a four-channel underwater acoustic recorder (SoundTrap ST4300HF, Ocean Instruments NZ); a three-dimensional-printed tetrahedral mount supporting four high-frequency hydrophones (HTI-99-HF, High Tech, Inc.); a motion datalogger (OpenTag, Loggerhead Instruments) to confirm that the device remained stationary; and a transponder to facilitate recovery (LRT, Sonardyne). A small (5 cm spacing) tetrahedral cluster of hydrophones was used to detect differences in time of arrival of sounds, and to estimate horizontal and elevation angles to echolocation clicks using methods similar to those described in [11]. However, whereas [11] used a tight array of tetrahedral clusters to determine three-dimensional locations, our study used a dispersed array to measure bearings at individual clusters.

**Figure 1.**
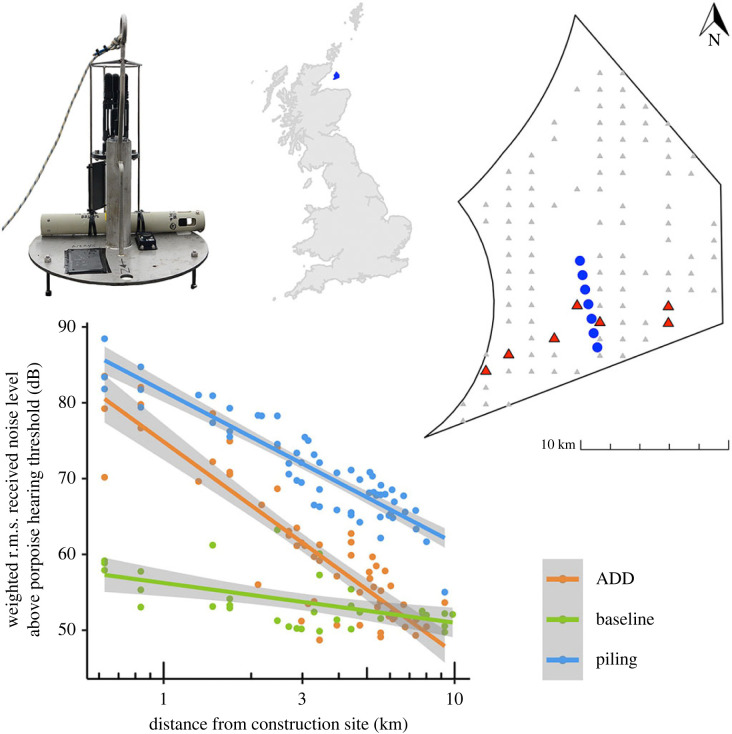
Top, directional hydrophone cluster, map of the UK showing the windfarm site (in blue) and windfarm detail showing locations of the clusters (blue circles) and sites (red triangles) with construction activity during acoustic recordings (grey triangles show the full turbine array). Bottom, mean received noise levels (dB above porpoise hearing threshold) on hydrophone clusters at different distances from construction during ADD mitigation, piling soft start and baseline periods.

A dispersed linear array of seven clusters was deployed within the Moray East Offshore Windfarm site (58°11′N, 2°43′W), Scotland between 21st August and 2nd September 2019 (figure 1). Individual SoundTraps recorded for 30 s every 2 min at a sample rate of 384 kHz.

### (b) Mitigation measures

Mitigation measures were required by regulators (see [15]), either when construction moved to a new turbine site or when there was a break in piling of longer than six hours at the same site. During our deployment, foundations were piled at seven turbine sites, 0.6–9.3 km from individual hydrophone clusters (figure 1). This resulted in ten mitigation events, each consisting of a 6–15-min period of acoustic deterrent device (Lofitech AS, Leknes, Norway; see [15,16] for signal characteristics) use and a 20-min piling soft start (electronic supplementary material, table S1). We compared porpoise responses during the ADD operation and the first 15 min of piling soft start with baseline data on directional movements from seven periods with no construction activity due to weather or mechanical breakdown (electronic supplementary material, table S1).

### (c) Noise characterization

Recordings on each hydrophone cluster were processed using PAMGUARD software [17] to determine noise levels as received on the recorders from the ADD, the piling soft start and baseline periods. The initial 5–6 pile strikes of piling soft starts were identified on acoustic recordings, and engineering records were used to correct any time drift. Received rms sound pressure levels were determined for 5 s intervals and frequency weighted with the harbour porpoise audiogram [18]. To characterize received noise levels during mitigation activities, received levels were calculated at each hydrophone cluster for each mitigation event and plotted against distance from the construction site.

### (d) Click classification and bearing determination

Porpoise clicks are high-frequency, narrow-band and can be readily distinguished from other transient sounds. The PAMGUARD click detector was configured to classify porpoise clicks as: click; echo; reflection; and buzz click (see electronic supplementary material, Porpoise Click Detection). Only clicks and buzz clicks were used in further analyses. To further screen weak or distant detections from the dataset, porpoise clicks occurring less than 4 min apart were classified as belonging to the same porpoise encounter and encounters with fewer than 5 clicks were excluded from further analyses to exclude false positive detections.

Horizontal angles to clicks were estimated as described in [11]. A second click detector in PAMGUARD was configured to detect and measure bearings to the lower-frequency piling noises. These bearings were compared with the known piling locations to determine the orientation of the clusters and thus orientate the bearings of detected porpoise clicks from each cluster. Accelerometer data (heading, pitch and roll) were inspected to confirm that the clusters remained stationary and data were excluded following any sudden movements (electronic supplementary material, table S2). To examine directionality of porpoise movements, we calculated the difference in the circular median bearing [19] to porpoise detections in each second and the bearing to the noise source from that hydrophone cluster. Values close to 0° represent porpoise clicks detected in a direction directly toward the construction site (see §3). Due to the highly directional nature of porpoise clicks, these are consistent with porpoises swimming directly away from the noise source (see electronic supplementary material, Angle of arrival; electronic supplementary material, figure S1). The distribution of differences in bearings was tested initially for uniformity, and then for uniformity against a unimodal alternative with a specified mean direction of 0° using Rayleigh tests in R [19,20]: to verify that test results were robust to the failure to account for the dependence structure of the data, data were also analysed by encounter (electronic supplementary material, table S5).

## 3. Results

### (a) Noise characterization

The duration of individual SoundTrap recordings varied between six and 11 days (electronic supplementary material, table S2). During baseline periods, mean received noise levels were relatively consistent across the array but decreased slightly with distance from the construction site (figure 1). We attribute slightly elevated background noise levels closer to the construction site to noise from construction vessels (figure 1). Mean received noise levels during ADD use and piling soft start declined with range (figure 1). Noise levels during ADD use approached baseline within 5 km, whereas piling soft starts remained at least 6 dB above background across the extent of our array and all received noise levels were significantly (greater than 50 dB) above the porpoise hearing threshold.

### (b) Movements during baseline periods

**Figure 2.**
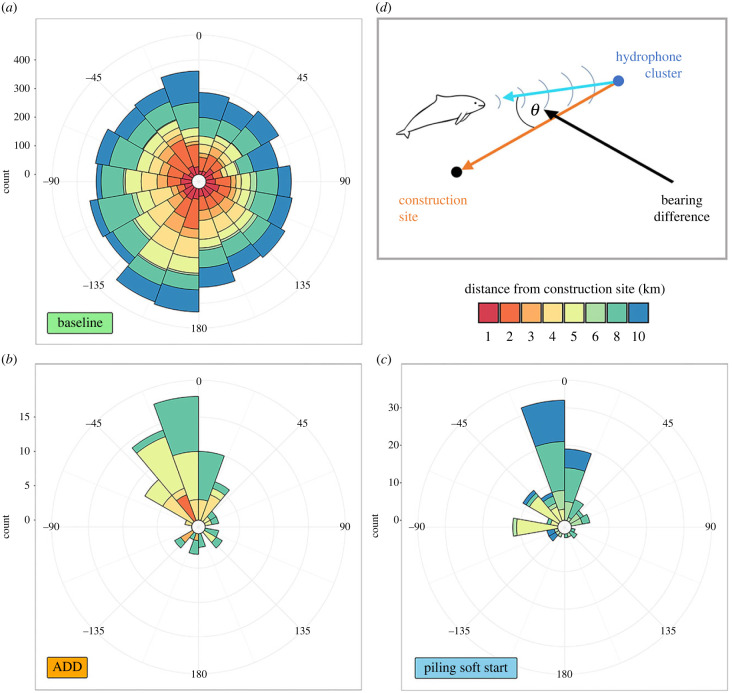
Harbour porpoise movements relative to the sound source during (*a*) baseline periods, (*b*) ADD mitigation and (*c*) piling soft start, and (*d*) schematic diagram illustrating how directionality of movement relative to the sound source was calculated for individual hydrophone clusters: bearings around 0° are indicative of evasive responses. Plots are circular histograms of the difference between the circular median bearing to porpoise detections each second and the bearing to the construction site.

During the baseline periods, there were 5925 s (0.46% of the time) during which porpoises were detected on any single hydrophone cluster (electronic supplementary material, table S3). These porpoise detections were distributed in all directions (figure 2*a*) and, although they were weakly directional (electronic supplementary material, table S4; Rayleigh test: *R* = 0.074, *p* < 0.001), they showed no departure from uniformity against an alternative with a specified mean direction of 0° (Rayleigh test: *R* = −0.052, *p* = 1.00).

### (c) Evasive responses to mitigation measures

During the deployment of mitigation measures, individual hydrophone clusters were between 0.6 and 9.3 km from the construction site (electronic supplementary material, table S3). The ADD was deployed for a total of 1.4 h while the hydrophone cluster array was *in situ*, during which there were 75 s (0.25% of the time) with porpoise detections (figure 2*b*; electronic supplementary material, table S3). In the 2.5 h of piling soft start, there were 112 s with porpoise detections (0.21% of the time) on the hydrophone clusters during the initial 15 min (figure 2*c*; electronic supplementary material, table S3). These sample sizes were insufficient to explore variation in evasive responses with distance. Nevertheless, by pooling all directional data within the range of distances studied, porpoise movements showed a strong directional response away from the sound source during ADD use and piling soft start when compared to baseline (electronic supplementary material, table S4; table S5; figure 2*b* and *c*; electronic supplementary material, figure S2). The null hypothesis of uniformity was rejected (Rayleigh test: ADD, R = 0.573, *p* < 0.001; piling, *R* = 0.626, *p* < 0.001) and the distribution of bearings to porpoise detections relative to the sound source, for both mitigation activities, was consistent with an alternative hypothesis with a specified mean direction of 0° (Rayleigh test: ADD, *R* = 0.564, *p* < 0.001; piling, *R* = 0.592, *p* < 0.001).

## 4. Discussion

We successfully demonstrated directional movement responses of harbour porpoises to mitigation measures prior to piling during construction of an offshore windfarm. The portable system developed here removes the dependence on existing infrastructure, extending the application of passive acoustic methods developed by [6] and [11] and providing opportunities to study responses of mobile, cryptic or rare species in specific underwater locations at particular times.

A limitation of using PAM is that if animals respond to disturbance by reducing vocalization rates some of their responses may not be detected (e.g. [21]). Therefore, although detection rates, expressed as the percentage of time with porpoise detections, decreased from 0.46% to 0.25% and 0.21% for ADD use and the piling soft start respectively, these numbers cannot tell us definitively whether there were fewer animals present, or whether they had changed their vocalization behaviour. Directional hydrophone clusters, however, showed clearly that vocalizing porpoises responded by moving away from the noise source both during ADD use and during the mitigation piling soft start. However, as the ADD was activated before every piling soft-start, observed responsive movements during the soft-start could represent a prolonged flight response initially triggered by the ADD. Some studies of cetacean responses to construction at offshore windfarms have been unable to distinguish the relative contribution of mitigation measures, piling or construction vessels to observed cumulative responses (e.g. [7,9]). In addition, displacement has not been observed directly, instead being inferred from changes in broader-scale occurrence of either acoustic [7,9,22] or visual [23] detections in response to disturbance events. This demonstration of negative phonotaxis is key to establishing the efficacy of mitigation measures for reducing the risk of injury or death in the near-field zone [24,25]. Additionally, it validates, at least for vocalizing individuals, a key assumption of agent-based models for assessing the population consequences of anthropogenic disturbances [26]; i.e. that animals respond to disturbance by moving away from the sound source. Based on simulations presented in electronic supplementary material ('Angle of arrival as an indicator of swim direction' and figure S1), it would appear that animals are swimming very directly away from the sound source with little deviation to either side.

Piling noise is predominantly low frequency [15] and porpoise hearing is most sensitive at high frequencies [27]. Audibility of both the ADD and piling would have been dependent both on environmental conditions [28] and on the level above the porpoise hearing threshold [8]. When filtered using the harbour porpoise audiogram and compared to both background noise and the porpoise hearing threshold, our data indicate that the sound of the piling soft start was likely to be audible to a range of at least 10 km. The ADD would have appeared quieter than the piling soft start, but likely would still have been audible to a porpoise to at least 4 km. However, due to the complex nature of the noise sources and known variation in audibility of signals as a function of their duration [18], we are unable to say with any precision just how far away the signals would have been audible to free-swimming animals.

Observed responsive movements away from ADD sources during the construction of an offshore windfarm (figure 2) are consistent with previous studies that conducted experimental field trials using the Lofitech ADD [16,29,30] or simulated Lofitech sounds [31]. However, the studies of cetacean responses relied on visual observations and all lost visual contact with some focal animals at or shortly after the time of exposure [16,30,31]. While experimental exposures of tagged animals provide opportunities to assess longer-range responses, the probability of tagged individuals occurring within specific locations during particular disturbance events can be extremely low for mobile species. Limited sample sizes precluded statistical analysis of variation in evasive responses with distance, nevertheless inspection of figure 2 revealed the range of distances at which responses were observed. Using our portable acoustic system, evasive responses were observed at distances of up to 7 km during ADD use and 9 km (the maximum distance between hydrophone clusters and the construction site; electronic supplementary material, table S3) during the piling soft start, overcoming constraints posed by studies that rely on visual observations or tagging. Acoustic studies have shown deterrence effects due to the Lofitech ADD over a similar range of distances [22,32].

Our results demonstrate how these techniques can improve the evidence base required to assess the costs and benefits of alternative mitigation measures, whether these be different types of ADD that reduce far-field disturbance or alternative approaches such as technical noise abatement systems (see [15,33]). The importance of context in determining individual behavioural responses has become increasingly evident [34], making it challenging to incorporate multiple contextual factors into either predictions or management advice based on experimental studies of individual responses. Harris *et al.* [35] advocate the use of opportunistic exposure studies to collect data over more relevant spatial and temporal scales to validate experimentally derived relationships and predictions on the scale of behavioural responses to noise. While not overcoming all the challenges involved in carrying out such work in open marine systems (see electronic supplementary material, additional discussion), our hydrophone cluster system means that targeting opportunistic studies of specific offshore activities spatially and temporally is more feasible than ever. This system now provides opportunities to characterize responses to mitigation measures and other disturbance sources across a broader and more representative suite of habitats and contexts.

## Data Availability

The data and R code for this study are available through the Dryad Digital Repository: https://doi.org/10.5061/dryad.7h44j0zvq [36]. Additional data are provided in electronic supplementary material [37].

## References

[1] Hays GC et al. 2016 Key questions in marine megafauna movement ecology. Trends Ecol. Evol. **31**, 463-475. (10.1016/j.tree.2016.02.015) 26979550

[2] Katzner TE, Arlettaz R. 2020 Evaluating contributions of recent tracking-based animal movement ecology to conservation management. Front. Ecol. Evol. **7**, 519. (10.3389/fevo.2019.00519)

[3] Cagnacci F, Boitani L, Powell RA, Boyce MS. 2010 Animal ecology meets GPS-based radiotelemetry: a perfect storm of opportunities and challenges. Phil. Trans. R. Soc. B **365**, 2157-2162. (10.1098/rstb.2010.0107) 20566493 PMC2894970

[4] Sugai LSM, Silva TSF, Ribeiro Jr JW, Llusia D. 2018 Terrestrial passive acoustic monitoring: review and perspectives. BioScience **69**, 15-25. (10.1093/biosci/biy147).

[5] Van Parijs SM, Clark CW, Sousa-Lima RS, Parks SE, Rankin S, Risch D, Van Opzeeland IC. 2009 Management and research applications of real-time and archival passive acoustic sensors over varying temporal and spatial scales. Mar. Ecol. Prog. Ser. **395**, 21-36. (10.3354/meps08123)

[6] Malinka CE, Gillespie DM, Macaulay JDJ, Joy R, Sparling CE. 2018 First *in situ* passive acoustic monitoring for marine mammals during operation of a tidal turbine in Ramsey Sound, Wales. Mar. Ecol. Prog. Ser. **590**, 247-266. (10.3354/MEPS12467)

[7] Graham IM, Merchant ND, Farcas A, Barton TR, Cheney B, Bono S, Thompson PM. 2019 Harbour porpoise responses to pile-driving diminish over time. R. Soc. Open Sci. **6**, 190335. (10.1098/rsos.190335) 31312495 PMC6599776

[8] Tougaard J, Wright AJ, Madsen PT. 2015 Cetacean noise criteria revisited in the light of proposed exposure limits for harbour porpoises. Mar. Pollut. Bull. **90**, 196-208. (10.1016/j.marpolbul.2014.10.051) 25467877

[9] Brandt MJ, Dragon AC, Diederichs A, Bellmann MA, Wahl V, Piper W, Nabe-Nielsen J, Nehls G. 2018 Disturbance of harbour porpoises during construction of the first seven offshore wind farms in Germany. Mar. Ecol. Prog. Ser. **596**, 213-232. (10.3354/meps12560)

[10] Blackwell SB, Nations CS, McDonald TL, Thode AM, Mathias D, Kim KH, Greene Jr CR, Macrander AM. 2015 Effects of airgun sounds on bowhead whale calling rates: evidence for two behavioral thresholds. PLoS ONE **10**, e0125720. (10.1371/journal.pone.0125720) 26039218 PMC4454580

[11] Gillespie D, Palmer L, Macaulay J, Sparling C, Hastie G. 2020 Passive acoustic methods for tracking the 3D movements of small cetaceans around marine structures. PLoS ONE **15**, e0229058. (10.1371/journal.pone.0229058) 32469874 PMC7259614

[12] Jarvis SM, Morrissey RP, Moretti DJ, DiMarzio NA, Shaffer JA. 2014 Marine mammal monitoring on navy ranges (M3R): a toolset for automated detection, localization, and monitoring of marine mammals in open ocean environments. Mar. Technol. Soc. J. **48**, 5-20. (10.4031/MTSJ.48.1.1)

[13] Wijers M, Loveridge A, Macdonald DW, Markham A. 2021 CARACAL: a versatile passive acoustic monitoring tool for wildlife research and conservation. Bioacoustics **30**, 41-57. (10.1080/09524622.2019.1685408)

[14] Simmonds MP, Brown VC. 2010 Is there a conflict between cetacean conservation and marine renewable-energy developments? Wildl. Res. **37**, 688-694. (10.1071/WR10020)

[15] Thompson PM, Graham IM, Cheney B, Barton TR, Farcas A, Merchant ND. 2020 Balancing risks of injury and disturbance to marine mammals when pile driving at offshore windfarms. Ecol. Solut. Evid. **1**, e12034. (10.1002/2688-8319.12034)

[16] Brandt MJ, Höschle C, Diederichs A, Betke K, Matuschek R, Nehls G. 2013 Seal scarers as a tool to deter harbour porpoises from offshore construction sites. Mar. Ecol. Prog. Ser. **475**, 291-302. (10.3354/MEPS10100)

[17] Gillespie D, Mellinger DK, Gordon J, McLaren D, Redmond P, McHugh R, Trinder P, Deng XY, Thode A. 2009 PAMGUARD: Semiautomated, open source software for real-time acoustic detection and localization of cetaceans. J. Acoust. Soc. Am. **125**, 2547. (10.1121/1.4808713)

[18] Kastelein RA, Hoek L, de Jong CAF, Wensveen PJ. 2010 The effect of signal duration on the underwater detection thresholds of a harbor porpoise (*Phocoena phocoena*) for single frequency-modulated tonal signals between 0.25 and 160 kHz. J. Acoust. Soc. Am. **128**, 3211-3222. (10.1121/1.3493435) 21110616

[19] Agostinelli C, Lund U. 2022 R package 'circular': circular statistics (version 0.4-95). https://r-forge.r-project.org/projects/circular/.

[20] R Core Team. 2020 R: a language and environment for statistical computing. Vienna, Austria: R Foundation for Statistical Computing.

[21] Wisniewska DM, Johnson M, Teilmann J, Siebert U, Galatius A, Dietz R, Madsen PT. 2018 High rates of vessel noise disrupt foraging in wild harbour porpoises (*Phocoena phocoena*). Proc. R. Soc. B **285**, 20172314. (10.1098/rspb.2017.2314) PMC5829196 29445018

[22] Dähne M, Tougaard J, Carstensen J, Rose A, Nabe-Nielsen J. 2017 Bubble curtains attenuate noise from offshore wind farm construction and reduce temporary habitat loss for harbour porpoises. Mar. Ecol. Prog. Ser. **580**, 221-237. (10.3354/MEPS12257)

[23] Dähne M, Gilles A, Lucke K, Peschko V, Adler S, Krügel K, Sundermeyer J, Siebert U. 2013 Effects of pile-driving on harbour porpoises (*Phocoena phocoena*) at the first offshore wind farm in Germany. Environ. Res. Lett. **8**, 025002. (10.1088/1748-9326/8/2/025002)

[24] Southall BL et al. 2007 Marine mammal noise exposure criteria: initial scientific recommendations. Aquat. Mamm. **33**, 411-521. (10.1578/AM.33.4.2007.411)

[25] Southall BL, Finneran JJ, Reichmuth C, Nachtigall PE, Ketten DR, Bowles AE, Ellison WT, Nowacek DP, Tyack PL. 2019 Marine mammal noise exposure criteria: updated scientific recommendations for residual hearing effects. Aquat. Mamm. **45**, 125-232. (10.1578/am.45.2.2019.125)

[26] Nabe-Nielsen J, van Beest FM, Grimm V, Sibly RM, Teilmann J, Thompson PM. 2018 Predicting the impacts of anthropogenic disturbances on marine populations. Conserv. Lett. **11**, e12563. (10.1111/conl.12563)

[27] Kastelein RA, Bunskoek P, Hagedoorn M, Au WW, de Haan D. 2002 Audiogram of a harbor porpoise (*Phocoena phocoena*) measured with narrow-band frequency-modulated signals. J. Acoust. Soc. Am. **112**, 334-344. (10.1121/1.1480835) 12141360

[28] Branstetter BK, Sills JM. 2022 Mechanisms of auditory masking in marine mammals. Anim. Cogn. **25**, 1029-1047. (10.1007/s10071-022-01671-z) 36018474 PMC9617968

[29] Gordon J, Blight C, Bryant E, Thompson D. 2019 Measuring responses of harbour seals to potential aversive acoustic mitigation signals using controlled exposure behavioural response studies. Aquat. Conserv.: Mar. Freshw. Ecosyst. **29**, 157-177. (10.1002/aqc.3150)

[30] McGarry T, Boisseau O, Stephenson S, Compton R. 2017 Understanding the effectiveness of acoustic deterrent devices (ADDs) on minke whale (Balaenoptera acutorostrata), a low-frequency cetacean. Report by Offshore Renewables Joint Industry Programme (ORJIP) Project 4, Phase 2, Report No. RPS Report EOR0692. London, UK: The Carbon Trust.

[31] Mikkelsen L, Hermannsen L, Beedholm K, Madsen PT, Tougaard J. 2017 Simulated seal scarer sounds scare porpoises, but not seals: species-specific responses to 12 kHz deterrence sounds. R. Soc. Open Sci. **4**, 170286. (10.1098/rsos.170286) 28791155 PMC5541550

[32] Brandt MJ, Höschle C, Diederichs A, Betke K, Matuschek R, Witte S, Nehls G. 2013 Far-reaching effects of a seal scarer on harbour porpoises, *Phocoena phocoen*a. Aquat. Conserv.: Mar. Freshw. Ecosyst. **23**, 222-232. (10.1002/aqc.2311)

[33] Juretzek C, Schmidt B, Boethling M. 2021 Turning scientific knowledge into regulation: effective measures for noise mitigation of pile driving. J. Mar. Sci. Eng. **9**, 819. (10.3390/jmse9080819)

[34] Ellison WT, Southall BL, Clark CW, Frankel AS. 2012 A new context-based approach to assess marine mammal behavioral responses to anthropogenic sounds. Conserv. Biol. **26**, 21-28. (10.1111/j.1523-1739.2011.01803.x) 22182143

[35] Harris CM et al. 2018 Marine mammals and sonar: dose-response studies, the risk-disturbance hypothesis and the role of exposure context. J. Appl. Ecol. **55**, 396-404. (10.1111/1365-2664.12955)

[36] Graham IM, Gillespie D, Gkikopoulou KC, Hastie GD, Thompson PM. 2023 Data from: Directional hydrophone clusters reveal evasive responses of small cetaceans to disturbance during construction at offshore windfarms. Dryad Digital Repository. (10.5061/dryad.7h44j0zvq) PMC9845968 36651028

[37] Graham IM, Gillespie D, Gkikopoulou KC, Hastie GD, Thompson PM. 2023 Directional hydrophone clusters reveal evasive responses of small cetaceans to disturbance during construction at offshore windfarms. Figshare. (10.6084/m9.figshare.c.6368803) PMC9845968 36651028

